# Supplementary figures and images for: Identifying potential natural inhibitors of Brucella melitensis Methionyl-tRNA synthetase through an in-silico approach
Source: PLoS Negl Trop Dis. 2022 Mar 21;16(3):e0009799. doi: 10.1371/journal.pntd.0009799 (PMC8970508; doi:10.1371/journal.pntd.0009799)

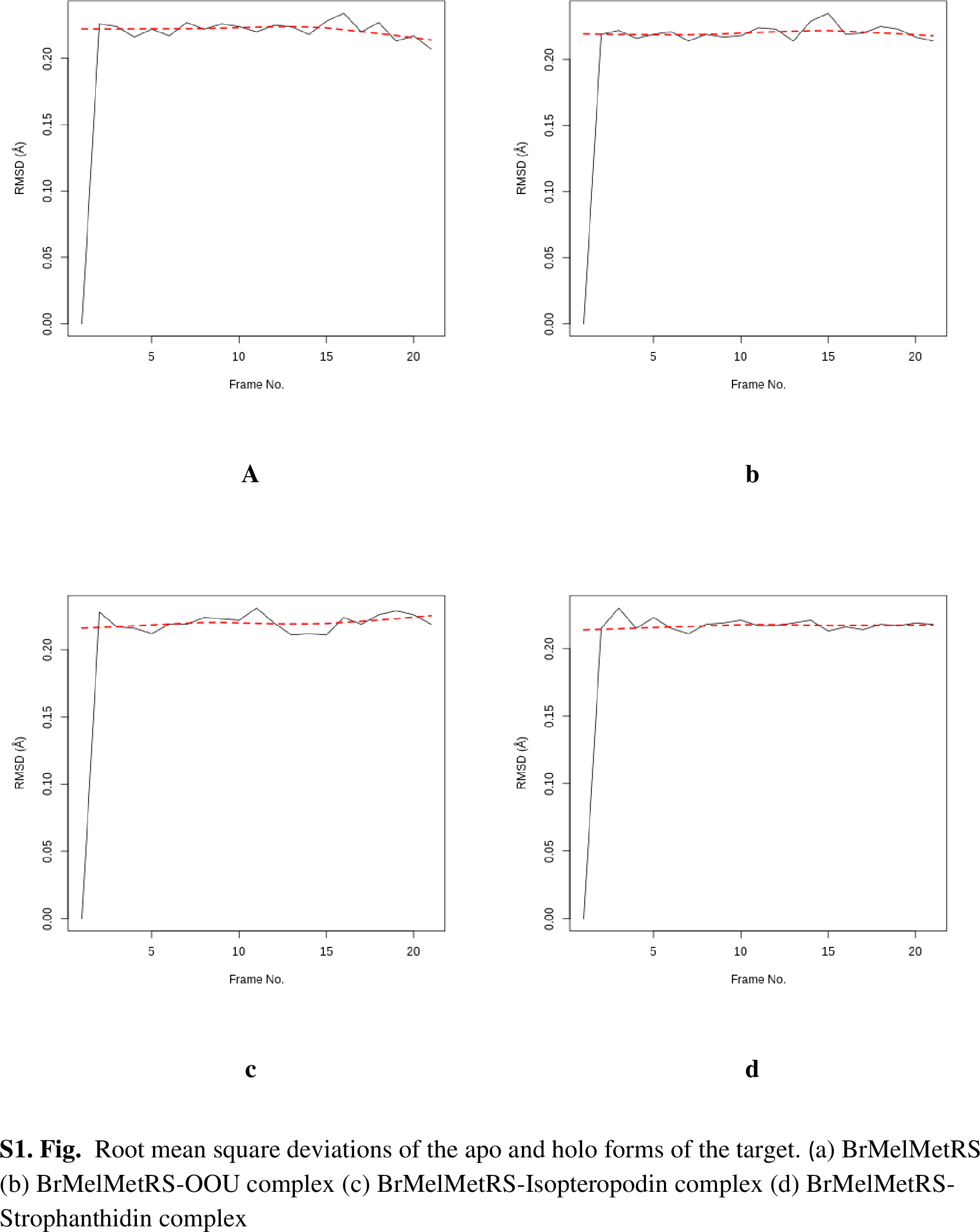

Supplement: S1 Fig — (TIF) [file pntd.0009799.s001.tif]

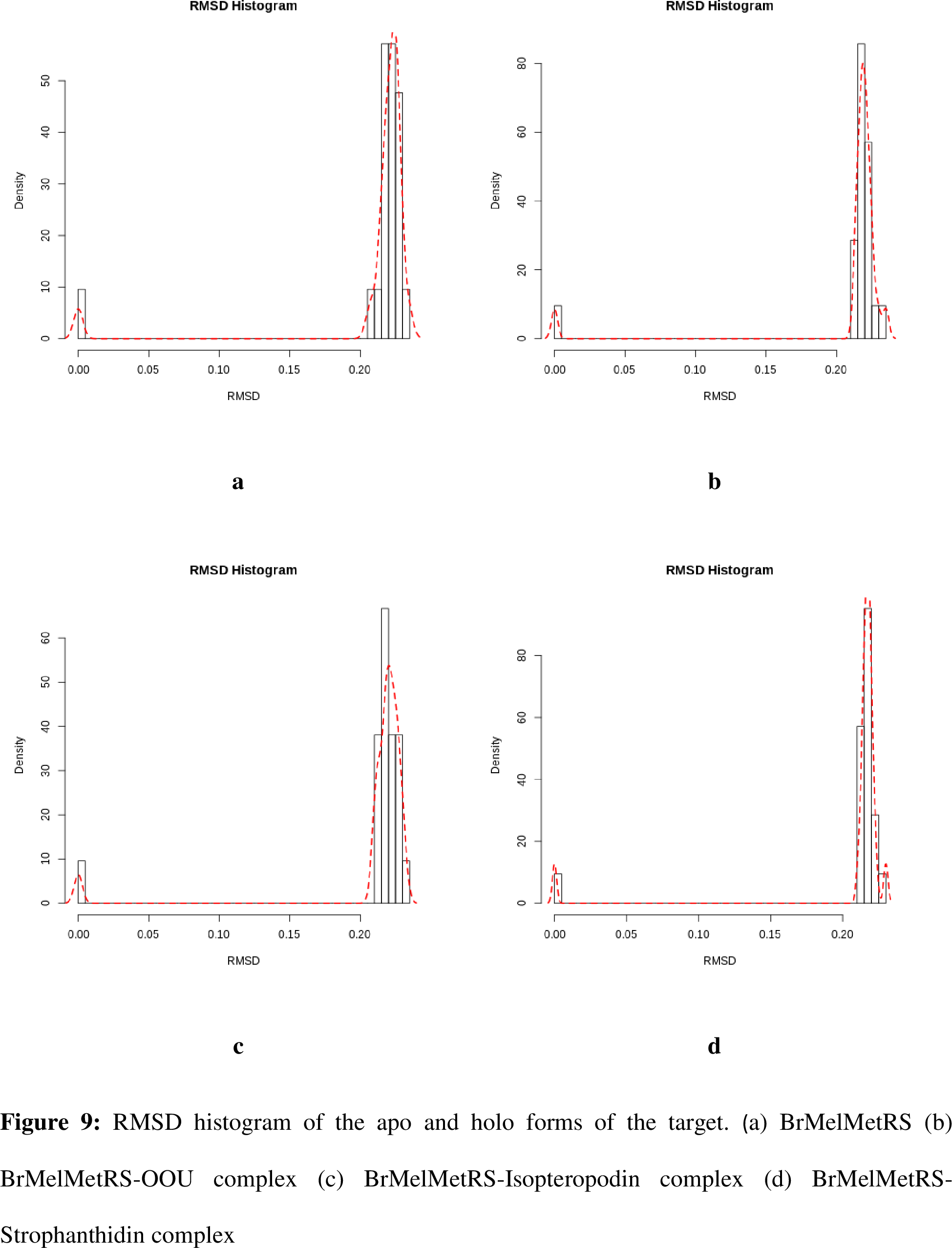

Supplement: S2 Fig — (a) BrMelMetRS (b) BrMelMetRS-OOU complex (c) BrMelMetRS-Isopteropodin complex (d) BrMelMetRS-Strophanthidin complex. (TIF) [file pntd.0009799.s002.tif]

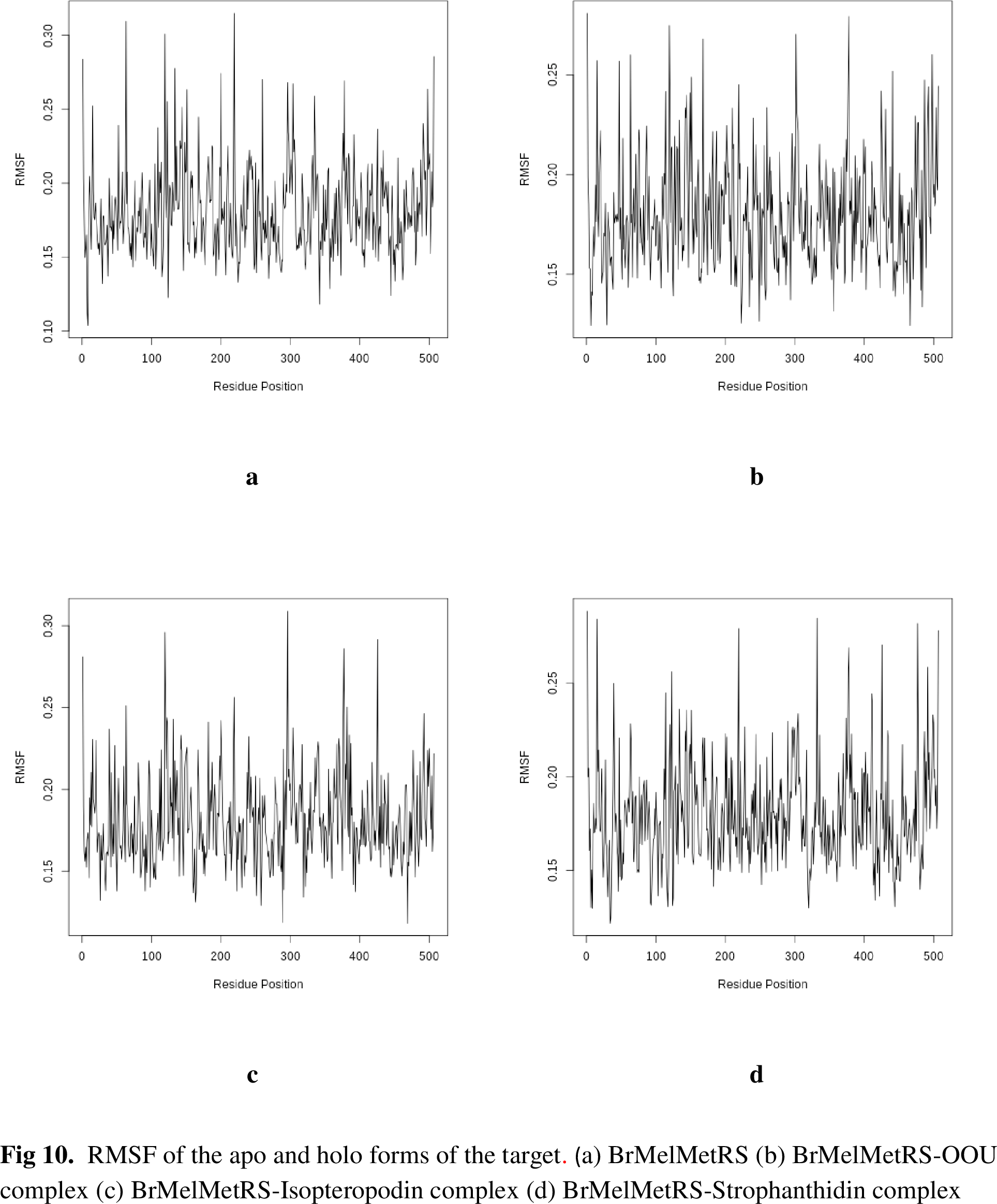

Supplement: S3 Fig — (a) BrMelMetRS (b) BrMelMetRS-OOU complex (c) BrMelMetRS-Isopteropodin complex (d) BrMelMetRS-Strophanthidin complex. (TIF) [file pntd.0009799.s003.tif]

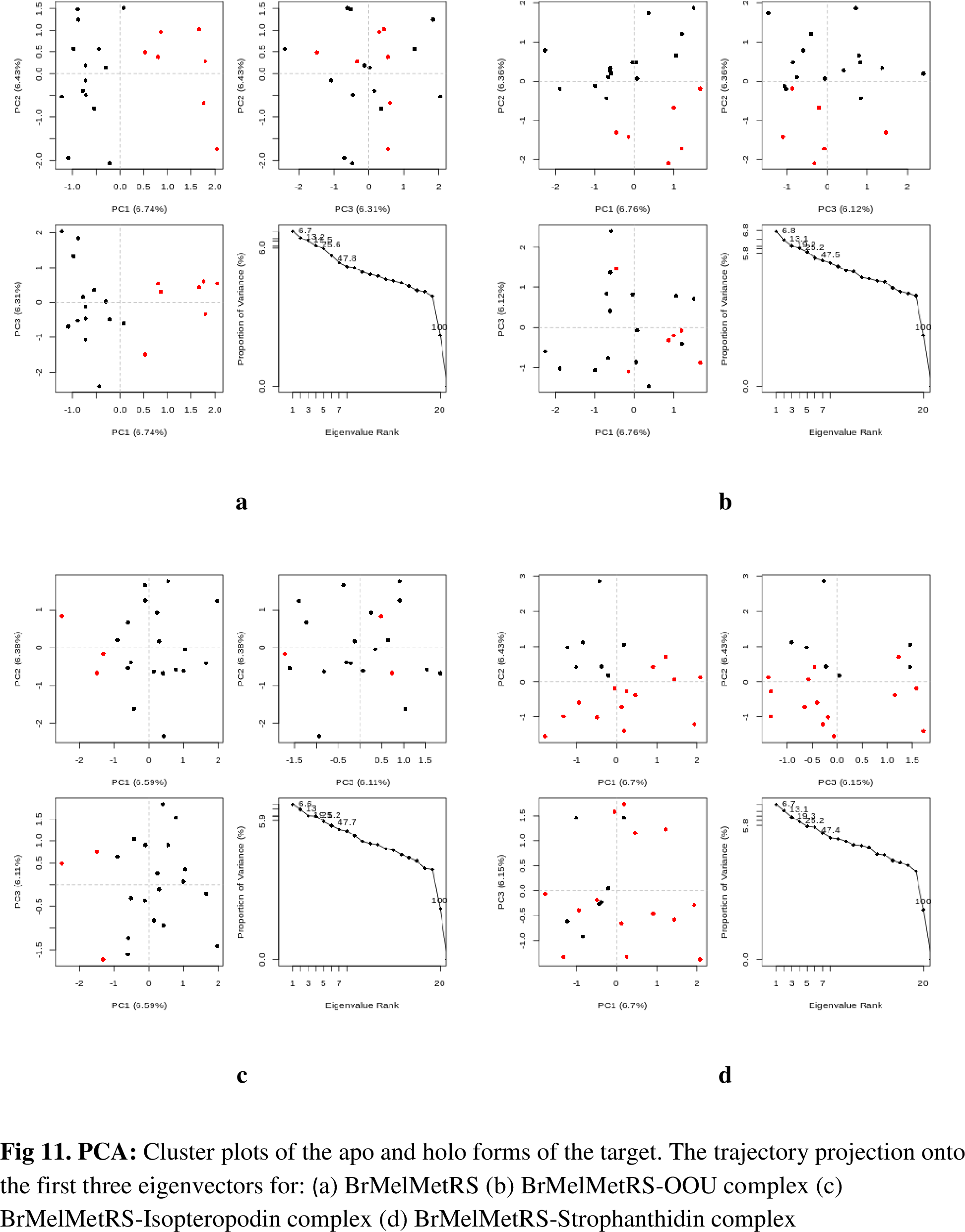

Supplement: S4 Fig — The trajectory projection onto the first three eigenvectors for: (a) BrMelMetRS (b) BrMelMetRS-OOU complex (c) BrMelMetRS-Isopteropodin complex (d) BrMelMetRS-Strophanthidin complex. (TIF) [file pntd.0009799.s004.tif]

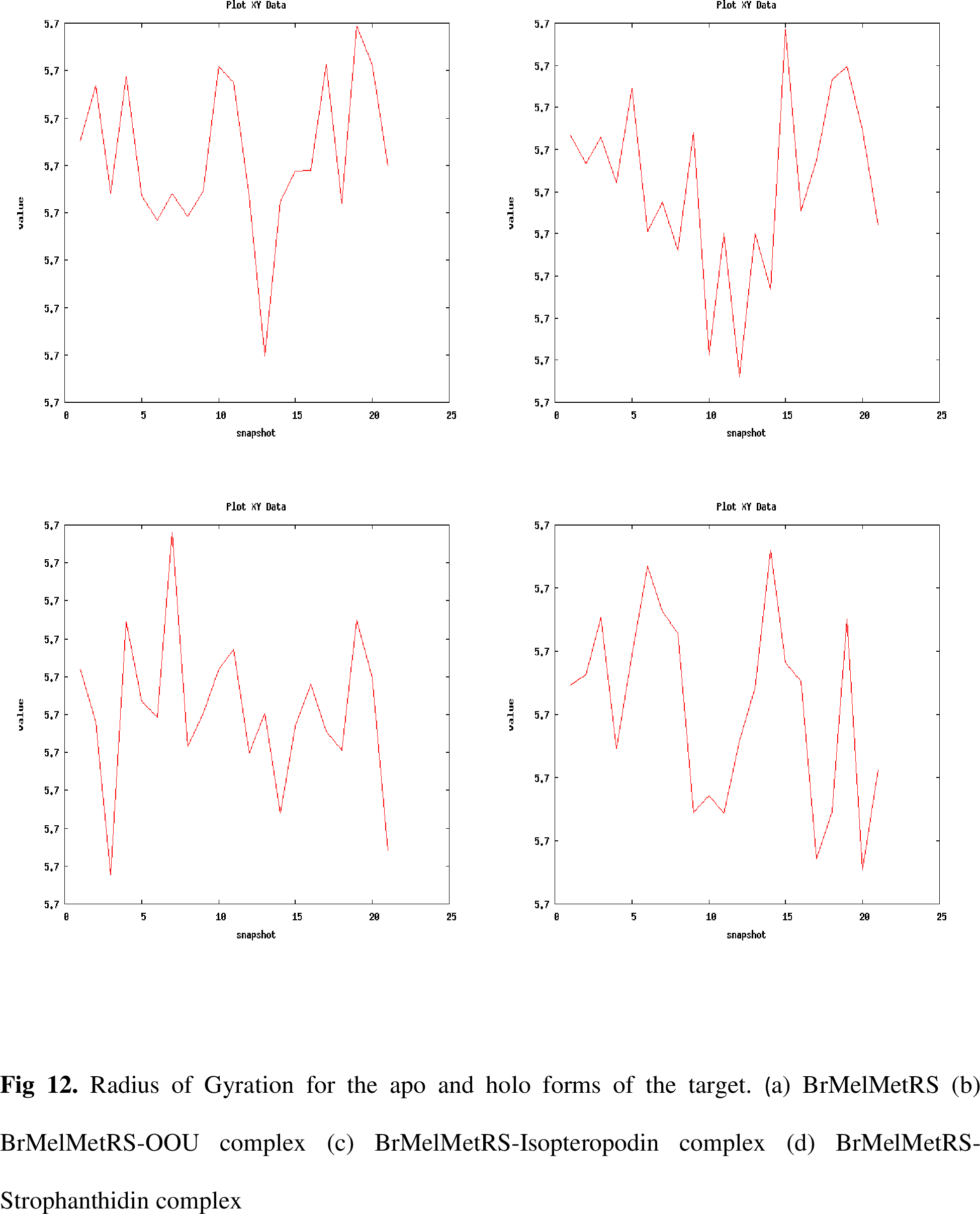

Supplement: S5 Fig — (a) BrMelMetRS (b) BrMelMetRS-OOU complex (c) BrMelMetRS-Isopteropodin complex (d) BrMelMetRS-Strophanthidin complex. (TIF) [file pntd.0009799.s005.tif]

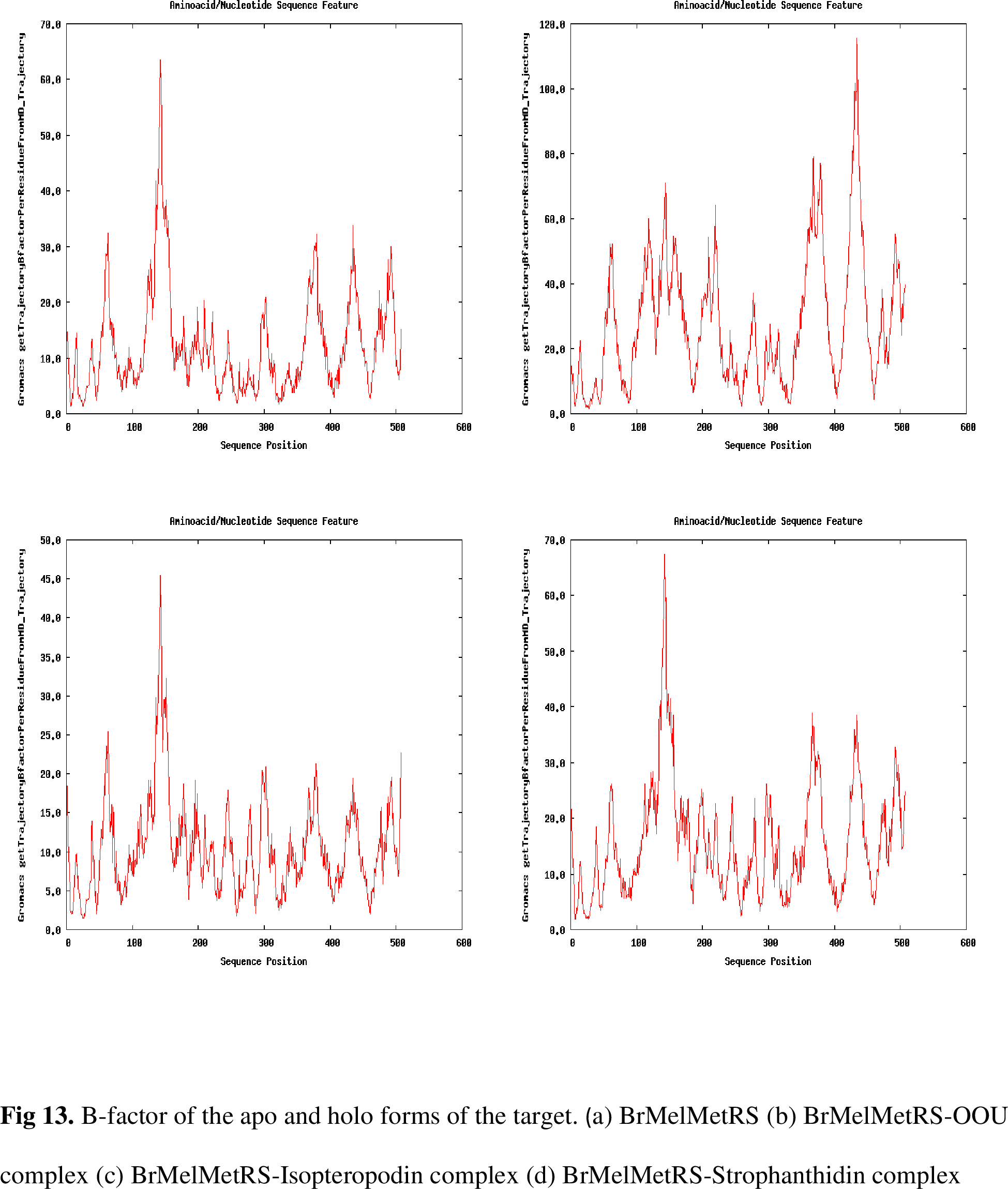

Supplement: S6 Fig — (a) BrMelMetRS (b) BrMelMetRS-OOU complex (c) BrMelMetRS-Isopteropodin complex (d) BrMelMetRS-Strophanthidin complex. (TIF) [file pntd.0009799.s006.tif]

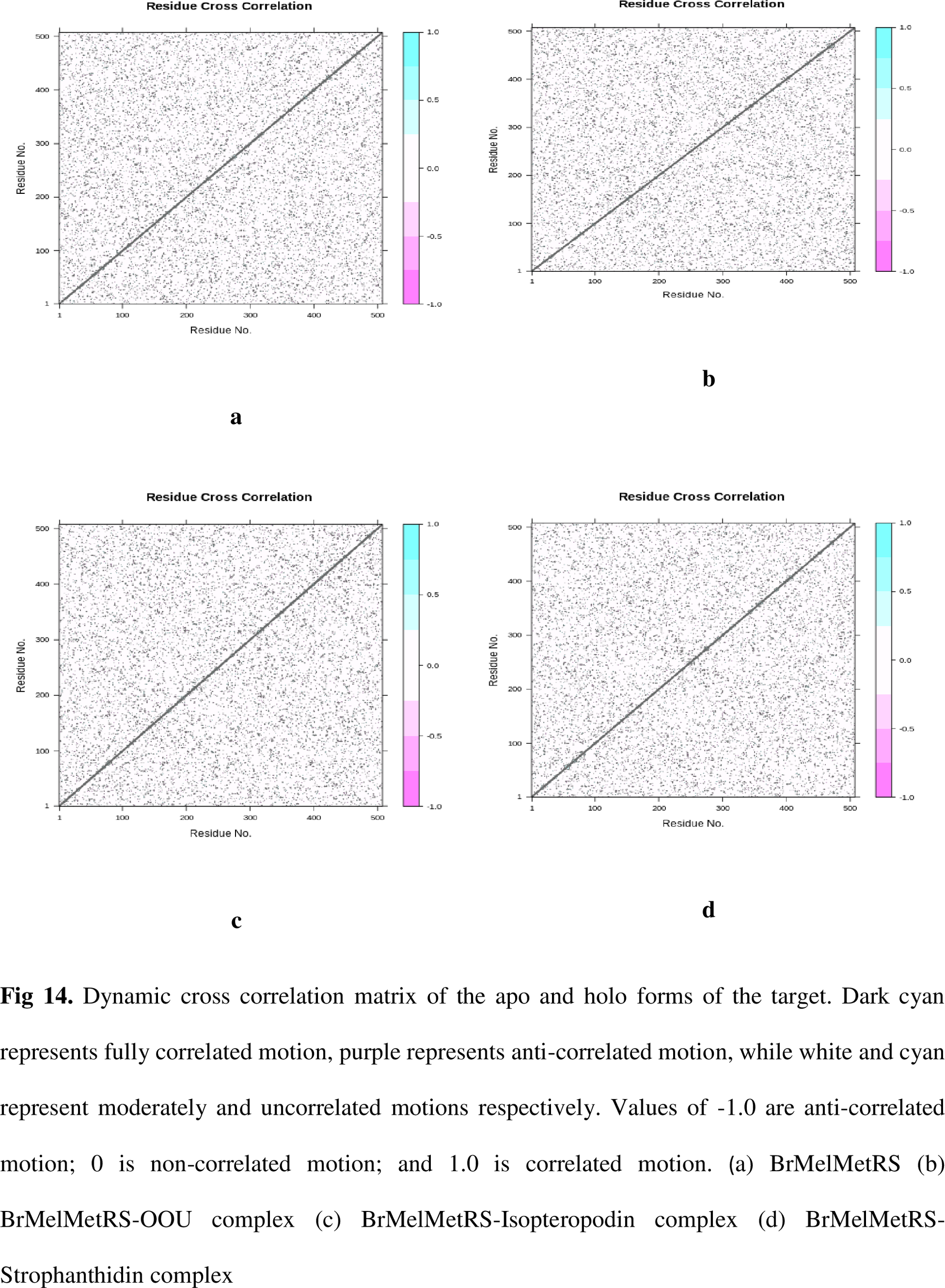

Supplement: S7 Fig — Dark cyan represents fully correlated motion, purple represents anti-correlated motion, while white and cyan represent moderately and uncorrelated motions respectively. Values of -1.0 are anti-correlated motion; 0 is non-correlated motion; and 1.0 is correlated motion. (a) BrMelMetRS (b) BrMelMetRS-OOU complex (c) BrMelMetRS-Isopteropodin complex (d) BrMelMetRS-Strophanthidin complex. (TIF) [file pntd.0009799.s007.tif]
