# Supplementary material for: Identifying potential natural inhibitors of Brucella melitensis Methionyl-tRNA synthetase through an in-silico approach
Source: PLoS Negl Trop Dis. 2022 Mar 21;16(3):e0009799. doi: 10.1371/journal.pntd.0009799 (PMC8970508; doi:10.1371/journal.pntd.0009799)
Supplement: Attachment — Submitted filename: Review comments.docx [file pntd.0009799.s009.docx]

**Introduction**

There is need to provide more literature on the potentials or use of medicinal plants in the control of infectious diseases, as a basis for the present investigation. Are the preset drugs of choice for brucellosis readily available and accessible? What is the magnitude of the drug resistance challenge?

**Methods**

How was safety of the compounds determined? Any LD_50_?

**Results**

Line 169: The first table cited is referenced “Table 4”, whereas, no table was previously cited.

**Discussion**

The discussion is filled with more of literature review than discussing the findings. I suggest reducing some of the literature review in this section. I recommend reducing the length of discussion section and make it more precise and succinct.

**Conclusion**

Concluding that the compounds could be used to treat human brucellosis is too ambitious. Exploring the compounds in animal models is required to validate this statement.
